# Efficient Strategy to Generate a Vectored Duck Enteritis Virus Delivering Envelope of Duck Tembusu Virus

**DOI:** 10.3390/v6062428

**Published:** 2014-06-20

**Authors:** Zhong Zou, Zhigang Liu, Meilin Jin

**Affiliations:** 1 State Key Laboratory of Agricultural Microbiology, Huazhong Agricultura University, Wuhan 430070, China; E-Mails: zz19841024@126.com (Z.Z.); 475589992@qq.com (Z.L.); 2 Key Laboratory of Development of Veterinary Diagnostic Products, Ministry of Agriculture, Wuhan 430070, China; 3 College of Veterinary Medicine, Huazhong Agricultural University, Wuhan 430070, China; 4 College of Life Science and Technology, AnQing Normal University, AnQing 246011, China

**Keywords:** duck enteritis virus, bacterial artificial chromosome, mating-assisted genetically integrated cloning, DTMUV: bivalent vaccine

## Abstract

Duck Tembusu virus (DTMUV) is a recently emerging pathogenic flavivirus that has resulted in a huge economic loss in the duck industry. However, no vaccine is currently available to control this pathogen. Consequently, a practical strategy to construct a vaccine against this pathogen should be determined. In this study, duck enteritis virus (DEV) was examined as a candidate vaccine vector to deliver the envelope (E) of DTMUV. A modified mini-F vector was inserted into the SORF3 and US2 gene junctions of the attenuated DEV vaccine strain C-KCE genome to generate an infectious bacterial artificial chromosome (BAC) of C-KCE (vBAC-C-KCE). The envelope (E) gene of DTMUV was inserted into the C-KCE genome through the mating-assisted genetically integrated cloning (MAGIC) strategy, resulting in the recombinant vector, pBAC-C-KCE-E. A bivalent vaccine C-KCE-E was generated by eliminating the BAC backbone. Immunofluorescence and western blot analysis results indicated that the E proteins were vigorously expressed in C-KCE-E-infected chicken embryo fibroblasts (CEFs). Duck experiments demonstrated that the insertion of the E gene did not alter the protective efficacy of C-KCE. Moreover, C-KCE-E-immunized ducks induced neutralization antibodies against DTMUV. These results demonstrated, for the first time, that recombinant C-KCE-E can serve as a potential bivalent vaccine against DEV and DTMUV.

## 1. Introduction

Duck Tembusu virus (DTMUV), family *Flaviviridae*, genus *Flavivirus*, is a newly emerging pathogen related to significant egg drop syndrome in laying ducks since 2010 [1]. In addition to egg drop syndrome, acute anorexia, retarded growth and neurological dysfunction are also presented by DTMUV-infected young ducks. The disease follows a very acute course with a morbidity of 90% to 100% and a mortality of 10% to 30% [2].

As a member of the family, *Flaviviridae*, DTMUV is a single-stranded and positive-sense RNA virus, a length of 10,990 nucleotides. The whole open reading frame (ORF) encodes three structural proteins: the envelope (E); the core (C); and membrane (prM) proteins. The ORF also encodes seven non-structural (NS) proteins (NS1-NS2A-NS2B-NS3-NS4A-NS4B-NS5). Among these proteins, the E protein is the principal antigenic determinant that serves important functions in receptor binding and membrane fusion [3]. Therefore, the E protein is a key target for vaccine and drug development. The emergence of DTMUV infection has seriously threatened the progression of the waterfowl industry in China. However, licensed DTMUV vaccine is currently unavailable. Hence, the development of a vaccine for ducks against DTMUV is critical for disease control.

Duck enteritis virus (DEV) belongs to anatid herpesvirus 1, and ducks, geese and swans are susceptible to it. The entire genome of DEV is approximately 160 kb and is composed of a unique long (UL), a unique short (US) and two inverted repeated sequences (IRS and TRS) [4]. A live C-KCE vaccine strain attenuated in embryonated chicken egg has been developed; it is also one of the safest and most effective duck vaccines available. The DEV vaccine strain is an ideal viral vector for an exclusive avian influenza virus vaccine in broilers [5,6]. Hence, C-KCE may be a promising candidate viral vector for developing other bivalent vaccines.

In this study, we established a bacterial artificial chromosome (BAC) of the C-KCE strain. In addition, the E gene of DTMUV was accurately inserted into the C-KCE genome based on the mating-assisted genetically integrated cloning (MAGIC) strategy. Our data reflected that the E gene inserted into the C-KCE genome was robustly expressed under the promoter of human elongation factor 1a (hEF1a). We further demonstrated that the insertion of the E gene exerted no adverse effect on the parental virus C-KCE. In addition, ducks immunized with the C-KCE-E vaccine produced DTMUV-specific antibodies. These ducks were also completely protected against virulent DEV. Overall, this study provides valuable information to establish bivalent live attenuated vaccines against DTMUV and DEV.

## 2. Materials and Methods

### 2.1. Virus Strain and Cells

The attenuated duck enteritis virus C-KCE vaccine strain, obtained from the China Institute of Veterinary Drugs Control, was propagated and titrated in chicken embryo fibroblast (CEF) cells (made by ourselves) propagated in Earle’s minimal essential medium (EMEM, Biochrom, Berlin, Germany), which was supplemented with 100 μg/mL penicillin, 100 μg/mL streptomycin and 10% fetal bovine serum (FBS) at 37 °C under a 5% CO_2_ atmosphere, and a virulent DEV strain isolated from Hubei province was propagated and titrated in duck embryo fibroblast (DEF) cells. A virulent DTMUV strain, which adapted to BHK21 through serially passage (GenBank ID: KJ489355), also was isolated from Hubei province.

### 2.2. Plasmids and Bacterial Strains

All the plasmids and *E. coli* strains were kindly donated by Dr. Lixin Ma. The mini-F plasmid pBlue-lox was maintained in *E. coli* strain DH10B-IS2 (umuC:araC-ParaBAD-I-Sce-I-FRT), which has been constructed in Lixin Ma’s lab and expresses enzyme *I-Sce*I induced by 0.2% w/v L-arabinose [7]. The plasmid, pML300, contained in DH10B-IS2 carries the *red* and *gam* recombinase gene stimulated by rhamnose and is unable to replicate when the host bacteria are grown at 42 °C [8]. DH10b was used for generating the transfer vector, pRThGA-E. DH10b, but not DH10B-IS2, would offer a trans-acting factor, π, which could support the conditional origin of replication from R6K, *ori*γ, which contained the donor vector, pRThGA1-E [9]. Plasmid pCAGGS-NLS/cre expressing Cre was described previously [10].

### 2.3. Generation of pBlue-lox-SORF3-US2-Amp Insertion Plasmid and of Donor Plasmid pRThGA1-E

Plasmid pBlue-lox-SORF3-US2-Amp contains two copies of the *Pac* I restriction site, an enhanced red fluorescent protein (RFP) gene and its cassette, two copies of the direct orientation 34-bp *Lox*p and two copies of the reverse complement, 18-bp *I-sce*I. For insertion of the 8.28-kb spanning BAC mini-F plasmid into the C-KCE genome, a 277-bp inter-genic region between the SORF3 and US2 genes was found to be suitable. Brieﬂy, the SORF3 upstream (partial) region and the inter-genic region were amplified as a 530-bp fragment using primers SORF3-F/SORF3-R (Table 1). The inter-genic region and downstream US2 region were then amplified as a 1.0-kb fragment using primers US2-F/US2-R (Table 1). The RFP gene under the control of the immediate early promoter of human cytomegalovirus (PHCMV) was amplified from pRTRA as a 1.8-kb fragment using primers Red-F/Red-R (Table 1). The three PCR products described above were used as the templates for “a ligation PCR” using primers US2-F/Red-R (Table 1); then, a 3.3-kb fragment was cloned into the *Sal* I/*Not* I sites of pBlue-lox, resulting in pBlue-lox-SORF3-US2. To increase the copy number of pBlue-lox, the ampicillin resistance gene replicon fragment was amplified from pcdna3.1 (+) with the primers, Amp-F/Amp-R (Table 1), and inserted into *Pac*I-digested pBlue-lox-gB-UL26 to obtain plasmid pBlue-lox-SORF3-US2-Amp.

**Table 1:** Primers used for generating pBAC-C-KCE, donor plasmid pRThGA and identification of the pBAC-C-KCE-E.

Purpose and Primer	Sequence (5’→3’)	Sequence Designation, Restriction Enzyme Site and Introduction Sequence
E Gene Clone ^a^		
E-1F	aaa**ggatcc**atgttcagctgtctggggatgcag	*BamH* I site (bold)
E-1R	aag**gaattc**ggcattgacatttactgccaggaa	*EcoR* I site (bold)
BAC Insertion ^b^		
US2-F	aaa**gtcgac**ataacttcgtatagcatacattatacgaagttatcacgtcttcccgcgaggcc	*Sal* I site (bold), *Lox* p sequence (underline)
US2-R	**ttaattaa**cgcggacaaaacgacgattac	*Pac* I site (bold)
SORF3-F	gtaatcgtcgttttgtccgcg**ttaattaa**tgaaaaagacggcggtacaat	*Pac* I site (bold)
SORF3-R	aagaatgcattcggcctgg	
Red-F	accagccagctgcgcttgctcgtggggtgtggtgcttttggt	
Red-R	tcga**gcggccgc**tagggataacagggtaatccccaccttatatattctttcccaccctcgaagagcgttc	*Not* I site (bold), *I-isce* I sequence (underline)
Amp-F	aaa**ttaattaa**ggggataacgcaggaaagaac	*Pac* I site (bold)
Amp-R	aaa**ttaattaa**acgtcaggtggcacttttcg	*Pac* I site (bold)
Modification pRThGA ^c^		
pCA-*I-Sce*I-H1-F	aaa**tagggataacagggtaat**gttgagcctttttgtggagtgggttaaattgtactagcgcgtttcgctttgcagtacatctacgtattagtcatcgctatta	*I-isce* I sequence (bold), Homology arm H1 (underline)
pCA-*I-Sce*I-H2-R	aaa**tagggataacagggtaat**tagcatgcataacttcgtataatgtatgctatacgaagttatgcggccgc cacacaggaaacagctatgaccatgattac	*I-isce* I sequence (bold), Homology arm H2 (underline)
Amp t-*I-Sce*I-F	aaa**attaccctgttatcccta**cacgttaagggattttggtcat	*I-isce* I sequence (bold)
OriT-R6K-*I-Sce*I-R	aaa**attaccctgttatcccta**	*I-isce* I sequence (bold)
Identification E ^d^		
E-2F	atgttcagctgtctggggatgcag	
E-2R	ggcattgacatttactgccaggaa	

a. Primers used to the clone the E gene of DTMUV; b. Primers used for the construction of the BAC insertion vector; c. Primers used for verification of the E gene inserted into the pBAC-C-KCE base using the MAGIC strategy; d. Primers used for the identification of the E gene of DTMUV.

Fragments from human elongation factor 1a (hEF1a) promoter to BGH ploy A were amplified from vector pEF6-v5/his with the primers, pCA-I-SceI-H1-F/pCA-I-SceI-H2-R, flanked by 50-bp homology arms and *I-sce*I restriction sites. The fragment cut by *I-sce*I was ligated into the pRThGA vector (also cut by the same enzymes), resulting in the recombinant plasmid, pRThGA1. Genomic RNA of DTMUV was extracted from DTMUV virions. An RT-PCR fragment encoding the E gene was amplified using primers E1-F/E1-R (Table 1). A start codon was added. The PCR product was digested with *BamH I* and *EcoR I* and ligated to pRThGA, resulting in pRThGA-E.

### 2.4. Construction of a C-KCE BAC Clone

**Figure 1:**
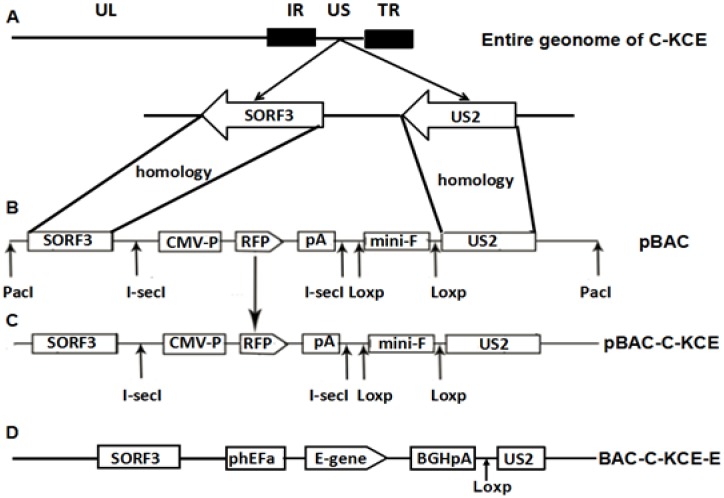
(**A**) The organization of the 158-kbp attenuated commercial duck enteritis virus (DEV) vaccine strain (C-KCE); (**B**) the organization of plasmid pBlue-lox-SORF3-US2-Amp digested by *Pac*I contains an enhanced red fluorescent protein gene and its expression cassette, two copies of the direct orientation 34-bp *Lox*p and two copies of the reverse complement, 18-bp *I-sce*I; (**C**) after homologous recombination, pBlue-lox-SORF3-US2-Amp was inserted into the genome of C-KCE with the red fluorescent protein as a selection marker; (**D**) the organization of the C-KCE-E.

Prior to the attachment and penetration of C-KCE virus at a multiplicity of infection (MOI) of 50, the virus was incubated to CEFs for 2 h at 37 °C; pBlue-lox-SORF3-US2-Amp linearized with *Pac* I (Figure 1B) was transfected by calcium phosphate precipitation as described earlier [11]. When the complete cytopathic effect was observed, the total supernatant was harvested; the infected virus was diluted and then plated on the fresh CEFs and overlaid with DMEM-FBS containing 0.5% methylcellulose. When red fluorescent plaques were observed, plaque-purification was carried out as described earlier [10] to obtain a fluorescent plaque population, termed vBAC-C-KCE. Circular viral DNA was extracted from CEFs by the method of Hirt [12]. A total 5 µg of genomic DNA was used to electroporate *DH10B-IS2* with 0.1-cm cuvettes under the following conditions: at 1.5 kV, a resistance of 200 Ω and a capacitance of 25 μF.

### 2.5. Generating the Recombinant pBAC-C-KCE-E Vector by MAGIC and Deleting the BAC Vector

*E. coli* DH10b containing the donor plasmid, pRThGA-E, was grown in LB broth containing 100 μg/mL ampicillin. The recipient strain, DH10B-IS2, containing the plasmid, pML300, and the recipient plasmid, pBAC-C-KCE, was grown in LB containing 50 μg/mL spectinomycin, 34 μg/mL chloramphenicol, 100 μg/mL streptomycin and 0.2% w/v glucose. The procedures of MAGIC were performed as described previously [8] with slight modifications (Figure 4). The positive clone was termed pBAC-C-KCE-E. To delete the BAC vector sequences, pC-KCE-BAC–E was co-transfected along with pCAGGS-NLS/cre into CEFs. The deleted BAC vector, termed the C-KCE-E virus, was purified by plaque.

### 2.6. Detection the Expression of E Protein

E protein expression in the C-KCE-E was evaluated by immunofluorescence (IFA) and western blot. The monoclonal antibodies (mAbs) against E and the polyclonal antibodies (pAbs) against gC were produced as described previously [13,14]. For IFA, the CEFs were infected at an MOI of 1 with C-KCE or C-KCE-E. The mAbs against E (made by us) was used as the primary antibodies. The secondary antibodies were ﬂuorescein isothiocyanate-conjugated goat anti-mouse IgGs (for E detection) (Santa Cruz). The CEFs nuclei were stained with 4’-6-diamidino-2-phenylindole (DAPI). Additionally, the cells were observed with a laser-scanning confocal microscope (Carl Zeiss, Heidenheim, Germany). The results were analyzed using the software, Image J (NIH, Bethesda, MD, USA). For western blot analysis, E expression was carried out in CEFs infected with C-KCE-E and C-KCE at an MOI of 1. mAbs against E, pAbs against gC (made by us) and mAbs against GAPDH (Santa Cruz, CA, USA) for the control were used as primary antibodies; goat HRP-conjugated anti-rabbit or anti-mouse IgGs were used as secondary antibodies. The bands were visualized using ECL detection reagents (Thermo, Waltham, MA, USA), according to the manufacturer’s instructions.

### 2.7. Stability and Growth Properties of the Recovered Virus C-KCE-E

To analyze the genetic stability of the C-KCE-E, the virus was grown on CEFs sequentially for 20 passages, and virus DNA was extracted and analyzed using primers E-2F/E-2R (Table 1). To compare the growth between C-KCE and C-KCE-E, assays of multi-step growth kinetics and measurements of plaque size were performed as described earlier [15].

### 2.8. Animal Experiments

Specific-pathogen-free (SPF) ducks were obtained from Harbin Veterinary Research Institute, China. Ducks were inoculated subcutaneously with 0.1 mL of PBS-diluted C-KCE or C-KCE-E and then intramuscularly challenged with lethal virulent DEV at different time points post-vaccination (p.v.). Ducks were observed daily for signs of disease and death after post-challenge (p.c.).

### 2.9. Neutralization Tests

At Weeks 2, 3 and 4 postvaccination, ducks that received the 10^5^ PFU C-KCE-E, 10^5^ PFU C-KCE or PBS were sacrificed humanely. Prior to the test, the serum samples obtained from the three groups were inactivated at 56 °C for 30 min. The test was performed using 9-day-old SPF chicken embryonated eggs as previously described [13].

### 2.10. Statistical Analysis

All experiments were reproducible and performed in triplicate. Statistical analyses were conducted by a one-way ANOVA test to compare the data of the difference groups using GraphPad Prism version 5.0 (GraphPad Software, La Jolla, CA, USA). *p*-values of <0.05 were considered statistically significant.

## 3. Results

### 3.1. Establishing a Full-Length C-KCE Clone Harboring Mini-F Plasmid Sequences

**Figure 2:**
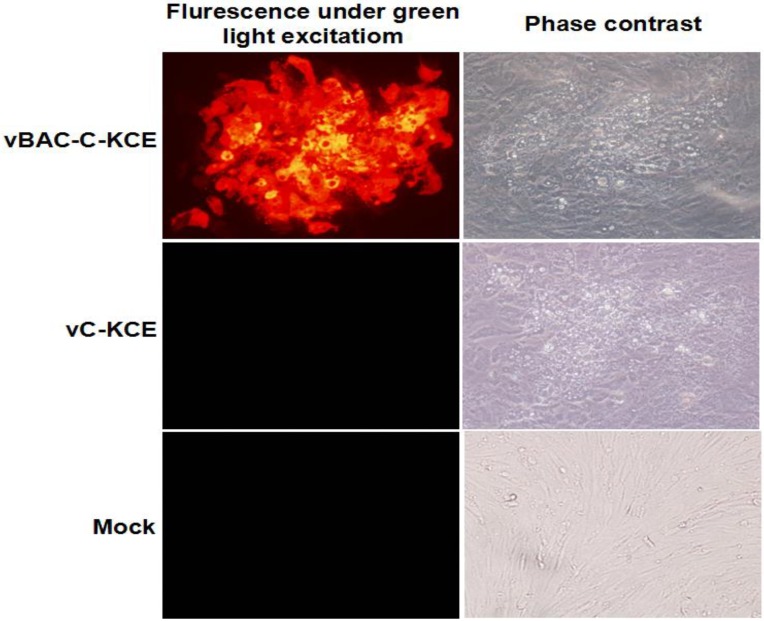
Plaques of recombinant vBAC-C-KCE-E, parental C-KCE virus and mock. Plaques are exposed to the excitation of 558 nm and the phase contrast.

**Figure 3:**
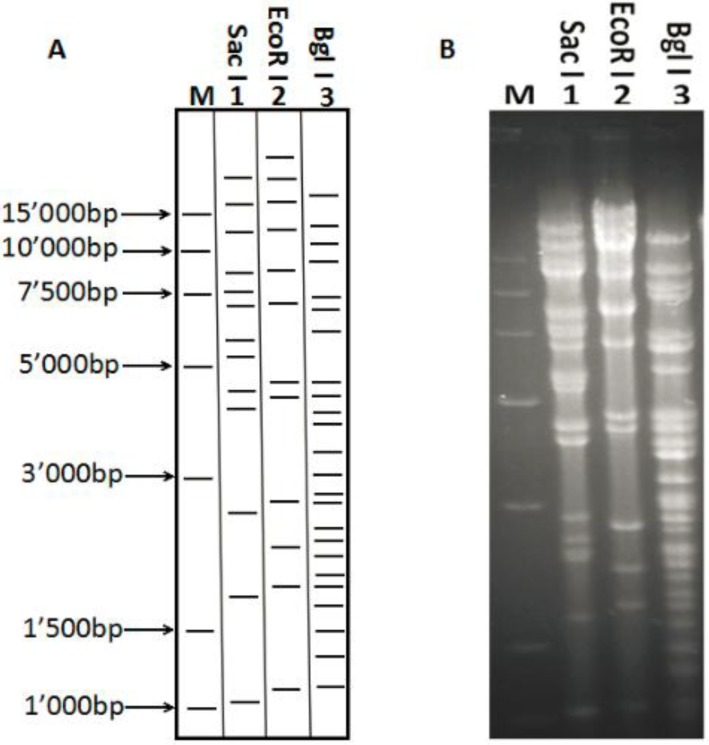
Restriction fragment analysis of pBAC-C-KCE. (**A**) The patterns corresponded exactly to the predictions based on the full C-KCE genome (KF263690.1). (**B**) pBAC-C-KCE was extracted and digested with *Bgl* I, *EcoR* I and *Sac* I and separated with a 0.8% agarose gel. The sizes of a molecular weight marker (M: DL15’000 bp, Transgen, beijing, china) were used.

To establish a full-length C-KCE clone, the BAC vector was inserted into a large junction of the SORF3 and US2 genes in the C-KCE genome (Figure 1A) homologous recombination. The resulting virus, vBAC-C-KCE, was plaque-purified based on the expression of red fluorescent protein (RFP) (Figure 2). The virus was passaged in chicken embryoblasts (CEFs) for 20 times to evaluate the genetic stability of the purified vBAC-C-KCE (Figure S1). The circular viral DNA from the vBAC-C-KCE-infected CEFs was extracted and used to transform *E. coli* strain DH10B-IS2. Three chloramphenicol-resistant colonies of pBAC-C-KCE (Figure 1C) were randomly collected 36 h after plating of the electroporated cells. Moreover, restriction fragment length polymorphisms (RFLP) were determined to confirm that a full-length BAC-C-KCE clone was indeed generated. The DNA of BAC-C-KCE was isolated and digested with enzymes *Bgl* I, *EcoR* I and *Sac* I. The RFLP patterns were compared with the predictions (Figure 3A), which were based on the reference complete genome sequence of the C-KCE strain (GenBank ID:KF263690.1). The obtained *Bgl* I, *EcoR* I and *Sac* I pattern coincided with those of the predictions well (Figure 3B). All clones were isolated using the QIAprep miniprep kit and then transfected into CEFs. Five days after the transfection, both clones had evident cytopathic effects. The findings indicated that the BAC was inserted into the SORF3 and US2 junction region in a site-specific manner.

### 3.2. Rapid Generation of C-KCE-E Bivalent Live Vaccie

**Figure 4:**
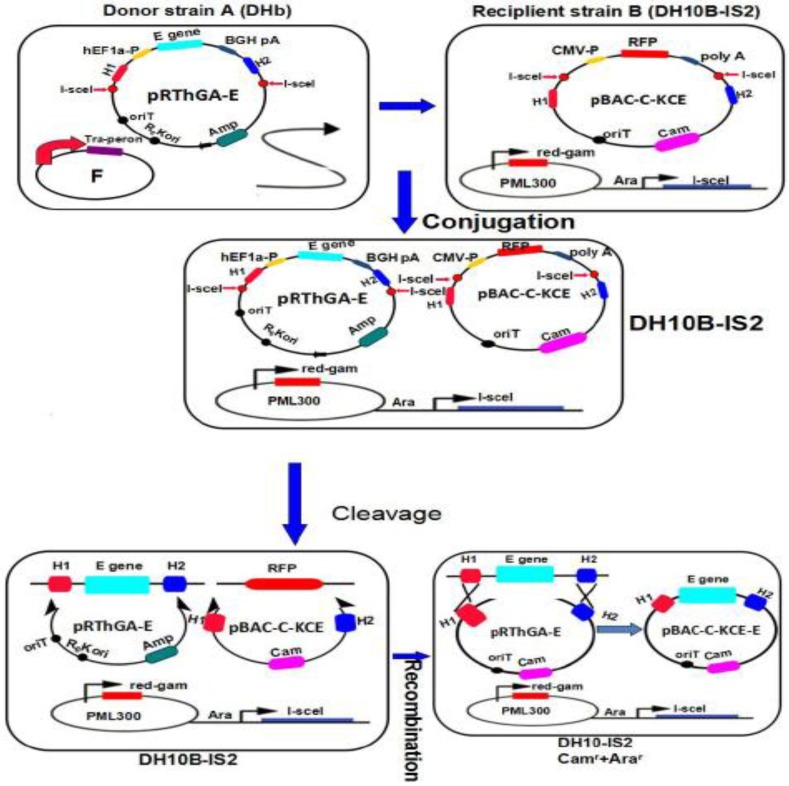
The steps for generating the pBAC-C-KCE-E base using the mating-assisted genetically integrated cloning (MAGIC) strategy. The donor and recipient plasmids were constructed as described in the text, then transformed into donor strain A (DH10b) and recipient strain DH10B-IS2. The DNA fragments of E plus its expression cassette in the donor vector (pRTHGA-E) and RFP and its expression cassette in the recipient vector (pBAC-C-KCE) were both cut down by an intron-encoding rare endonuclease *I-Sce*I stimulated by 0.2% w/v L-arabinose, and then, the recombination events were intermediated by the red and gam recombinase induced by 0.2% w/v rhamnose. Then, the recombinant pBAC-C-KCE-E was generated.

**Figure 5:**
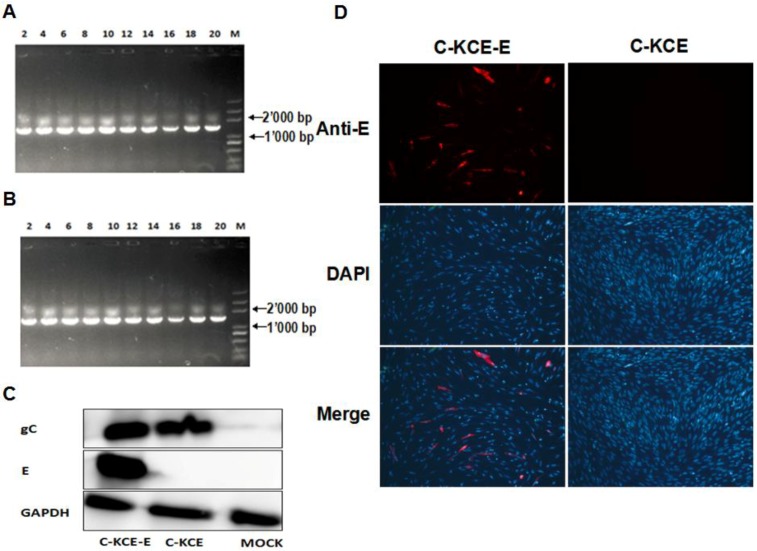
The efficiency of E gene insertion in the pBAC-C-KCE base using MAGIC and characterization of the recombinant C-KCE-E viruses. (**A**) Detection of the E gene insertion in pBAC-C-KCE by PCR. The marker used was DL2000. (**B**) Detection of the E gene insertion in the recombinant viruses by PCR. The numbers show the passages of C-KCE-E. The marker used was DL5000. (**C**) Confirmation of the expression of the E protein in C-KCE-E-infected chicken embryo fibroblasts (CEFs) by using immunoﬂuorescence. (**D**) Detection of the expression of E protein in C-KCE-E-infected CEFs by western blotting.

The E gene was inserted successfully into pBAC-C-KCE through the MAGIC strategy (Figure 4). Ultimately, the E gene was successfully inserted into pBAC-C-KCE. The red fluorescent gene, CMV promoter and ployA were entirely replaced by the E gene and its cassette. Hence, the E gene was expressed under the promoter of human elongation factor 1a (hEF1a). The colonies were randomly selected and screened through PCR using primers E2-F/E2-R (Table 1). An approximately 1.5-kb fragment was amplified from the positive recombinant plasmid, pBAC-C-KCE-E (Figure 5A). Among the randomly selected 24 colonies, 21 contained the expected recombinant plasmid, pBAC-C-KCE-E.

The efficiency of the recombination was 90%. BAC vector sequences were flanked by two direct orientation *Loxp* sequences. Moreover, pBAC-C-KCE-E was co-transfected with pCAGGS-NLS/cre to remove the BAC vector sequences. After the Cre-mediated removal of the BAC vector sequences, non-fluorescent plaques appeared and were collected. The resulting virus C-KCE-E (Figure 1D) without the BAC backbone was plaque-purified and then confirmed by PCR (data not shown). Our experimental results showed that the E gene can be inserted into pBAC-C-KCE by the MAGIC strategy. Moreover, the virus C-KCE-E, whose BAC backbone was eliminated, was successfully generated through *Cre/Lox*p-mediate recombination; only the 34 bp *Loxp* sequences were positioned in the C-KCE-E genome.

### 3.3. Biological Characterization and Stability of the Rescued C-KCE-E Recombinant Viruses

To evaluate the genetic stability and growth kinetics of C-KCE-E, the virus was grown on CEFs sequentially for 20 passages. The virus DNA was extracted and analyzed after with the primers, E2-F/E2-R (Table 1). The E gene in C-KCE-E was detected (Figure 5B). A multi-step growth kinetic assay was performed, and the plaque sizes were measured to compare the replication of C-KCE, vBAC-C-KCE and C-KCE-E. The growth kinetics of C-KCE-E and vBAC-C-KCE was similar to that of C-KCE (Figure S2A). The plaque sizes were also similar (Figure S2B). These results revealed that the E gene was stably inserted into the C-KCE genome and showed no adverse reaction on C-KCE replication *in vitro.* The expression of E protein was determined by western blot and IFA. As expected, cells infected with C-KCE-E reacted well, and strong signals were visualized using ECL detection reagents with mAb for E or pAb for UL23. By contrast, the parental virus C-KCE reacted well only when with pAb for UL23, and the uninfected cells did not react with both antibodies (Figure 5D). The results of IFA coincided with those of western blot. As shown in Figure 5C, diffused E expression was observed, indicating that the E protein was expressed in the C-KCE-E-infected CEFs. These results also implied that the E protein was robustly expressed during C-KCE-E replication.

### 3.4. Vaccine Efficacy against Lethal DEV Challenge in Ducks

**Figure 6:**
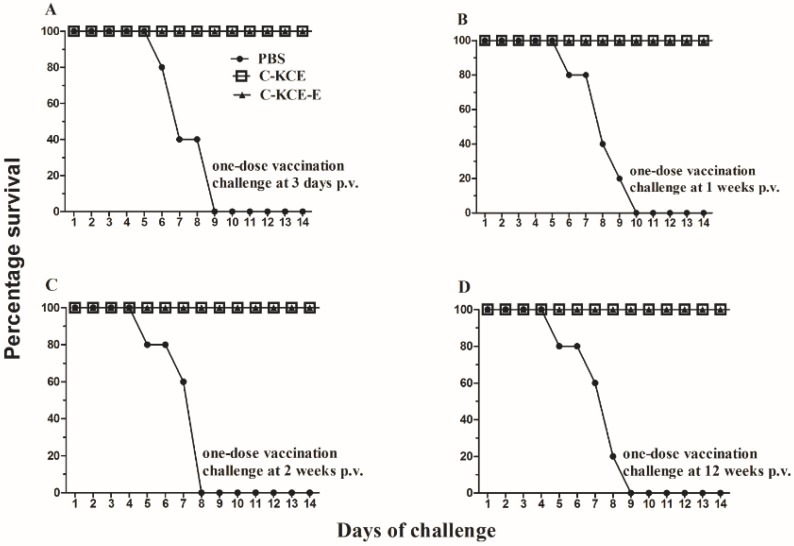
Protective efficacy of C-KCE-E against lethal DEV challenge. Groups of five ducks were inoculated subcutaneously with 10^5^ PFU of C-KCE-E, 10^5^ PFU of C-KCE or with PBS as a control, then intramuscularly challenged with 100-fold duck lethal dose (DLD_50_) virulent DEV at three days (**A**), one week (**B**), three weeks (**C**) or 12 weeks (**D**) postvaccination (p.v.), respectively. Ducks were monitored daily for 14 days after challenge.

Animal experiments were conducted to examine the effect of the inserted exogenous gene on the protective efficacy of the parental virus C-KCE and to evaluate the efficacy of the C-KCE-E vaccine against lethal DEV challenge. Ducks (five per group) were injected subcutaneously with 10^5^ PFU of C-KCE or C-KCE-E, whereas native control ducks were inoculated with PBS. The ducks were then challenged with a 100-fold 50% duck lethal dose (DLD_50_) of virulent DEV by intramuscular injection at 3 d and one, three and 12 weeks post-vaccination (p.v.), respectively. As shown in Figure 6, the ducks immunized with C-KCE or C-KCE-E survived regardless of the challenge with virulent DEV. Conversely, the PBS-inoculated ducks developed severe symptoms and succumbed to infection within 8–9 days. The protective efficacy of C-KCE-E and C-KCE against the lethal DEV challenge was not significantly different. These results demonstrated that the insertion of the E gene did not alter the protective efficacy of C-KCE.

### 3.5. Antibody Responses against DTMUV Induced by C-KCE-E Immunization in Ducks

**Figure 7:**
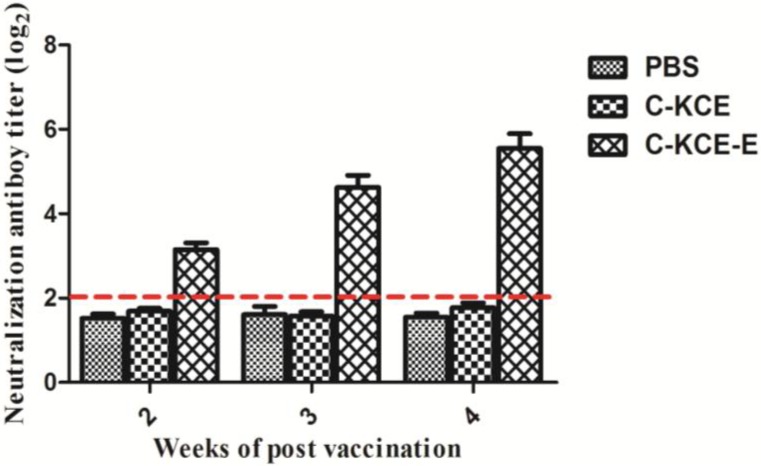
Antibody responses against duck Tembusu virus (DTMUV) induced by C-KCE-E immunization in ducks. Groups of five ducks were inoculated subcutaneously with 10^5^ PFU of C-KCE-E, C-KCE or PBS as a negative control. Sera were collected in a range from two to four weeks to detect the NT antibody against DMTUV in nine-day-old specific-pathogen-free (SPF) chicken embryonated eggs. NT antibody titers for ducks are expressed as a log_2_. Dotted lines indicate the thresholds for a positive response.

Three groups of ducks (five per group) were subcutaneously inoculated with C-KCE-E (10^5^ PFU), C-KCE (10^5^ PFU) or PBS (control) to evaluate whether or not C-KCE-E induces antibody responses against DTMUV. At two, three and four weeks p.v., serum samples were obtained weekly from all ducks to screen for the NT antibody, a marker of immunogenicity, against DTMUV. As shown in Figure 7, the NT antibody titers of the PBS- and C-KCE-inoculated groups were lower than 2^2^ and were considered negative. The NT antibody titers of the C-KCE-E-inoculated groups exceeded 2^3^ at two weeks p.v., reached 2^4^ at three weeks p.v. and continued to increase to 2^5^ ± 2^1^ at four weeks p.v. (Figure 7). These results demonstrated that C-KCE-E can induce antibody responses against DTMUV.

## 4. Discussion

The newly emerged duck Tembusu viral disease caused by DTMUV severely threatens the progression of the duck industry. Similar to the other members of the *Flaviviridae* family, DTMUV may also pose a potential threat to mammals, even including humans [16,17]. Close contact between humans and ducks or duck products is inevitable. Therefore, a vaccine against DMUV should be developed for potential public health concerns and for the duck industry.

Vaccination is the most effective method to control this pathogen. Currently, no licensed DTMUV vaccine is available. Only FX2010-180p attenuated in embryonated chicken egg can be utilized for a live attenuated vaccine candidate [18]. In this study, we established a BAC clone of the DEV attenuated strain C-KCE. The E gene of DTMUV was inserted into the C-KCE genome based on the MAGIC strategy. Although C-KCE-E contained an E gene and its cassette, the insertion had an adverse effect of this insertion on C-KCE replication, egress or viral pathogenesis in our assays. This study is the first to demonstrate the potential of C-KCE-E as a bivalent live attenuated vaccine against DTMUV and DEV.

BAC, which is the most popular platform to generate recombinant virus, has been extensively studied in many viruses. The BAC of C-KCE can be used to develop a bivalent vaccine, as well as to facilitate research on DEV pathogenesis. For instance, the DEV virulent strain 2085 is the first established infectious BAC to evaluate the function of the gC gene [10]. To establish a BAC clone of C-KCE, the BAC vector should be inserted into a large inter-genic region of the C-KCE genome. The full genome of C-KCE has been sequenced recently in our lab (GenBank ID:KF263690.1). The sequencing revealed that C-KCE and other herpesvirus type 1 share the similar molecular characterization in their genomes. The gene junction of SORF3 and US2 has been proven to be suitable for foreign gene insertion [19]. Therefore, the same location was selected for constructing the BAC of C-KCE. The expression of the neighboring genes could not be interfered at this junction. In addition to the BAC strategy, there are several other routine strategies that can be used to generate recombinant herpesvirus, such as cosmid. Compared with BAC, cosmid has limited capacity. Thus, inserting several immunogenic foreign genes to develop a polyvalent vaccine is difficult. More importantly, transfection of the cosmid vectors into target cells requires several rounds of homologous recombination to recover the virus; thus, accuracy is not guaranteed [20].

We accurately and efficiently inserted the E gene into pBAC-C-KCE based on the MAGIC method. To the best of our knowledge, the strategy described here is the first case to use MAGIC for the construction of recombinant DEV. The efficiency of the recombination can reach 90%, because the *red* and *gam* system is extremely highly efficient and because arabinose can function as a selection marker [7]. Utilizing MAGIC, we successfully constructed and obtained the recombinant C-KCE-E within two weeks of acquiring the E gene. Therefore, the approach is a highly-efficient, time-saving and labor-saving method for generating recombinant DEV.

Generally, the level of expression has two important determinants, including the inserted gene itself and its promoter [21]. Sonoda et al. compared the protective efficacy of the chicken β-actin promoter and the SV40 promoter for expressing the F gene of Newcastle disease virus inserted between the US10 of Marek's disease virus. They found that the chicken β-actin promoter performed better than the SV40 promoter and that the expression of F from the chicken β-actin promoter offered better protection against virulent MDV challenge [22]. Thus, we concluded that enhancing the expression level of the E gene is critical to inducing a higher level of protective neutralization antibodies against DTMUV. For this purpose, we chose the hEF1α promoter, driving a high-level of protein expression across a wide range of species and cell types. Indeed, the results of western blot and IFA manifested that the E protein was robustly expressed during C-KCE-E replication.

Neutralizing the antibody against DTMUV is the hallmark of protective immunity. As shown in the animal tests, C-KCE-E vaccination can induce DTMUV-specific antibodies at three weeks p.v. Considering that DTMUV challenge was not performed in this study, we were uncertain about the efficacy of the vaccine against a lethal DTMUV challenge in ducks. The DEV challenge test showed that the ducks immunized with C-KCE or C-KCE-E survived. Therefore, we concluded that the inserted gene did not alter the protective efficacy of the parental virus C-KCE.

In summary, we successfully developed one recombinant DEV delivering E of DTMUV. In our future studies, we will determine whether or not C-KCE-E can provide optimum protection against DTMUV infection in ducks. If the result is positive, C-KCE-E will be an extremely cost-effective option to control the devastating effect of DEV and DTMUV in ducks.

## 5. Conclusions

The bacterial artificial chromosome of an attenuated duck enteritis vaccine strain, C-KCE, was successfully established by inserting a modified mini-F vector into the gene junction between SORF3 and US2. The E gene of DTMUV was inserted into the C-KCE genome through mating-assisted genetically integrated cloning to produce the recombinant vector, pBAC-C-KCE-E. Our data reflected that the E gene inserted into C-KCE genome can be robustly expressed under the promoter of hEF1a. We also demonstrated that the insertion of the E gene exerted no adverse effect on C-KCE replication *in vitro* and did not alter the pathogenicity and immunogenicity of the parental virus C-KCE. In addition, ducks immunized with the C-KCE-E vaccine produced H5 DTMUV-specific antibodies. Our findings highlighted the potential of a BAC-C-KCE-vector-based delivery system, which would be widely utilized in the poultry industry.

## Supplementary Files

Supplementary File 1 Supplementary Materials (PDF, 295 KB)

## References

[1] Su J., Li S., Hu X., Yu X., Wang Y., Liu P., Lu X., Zhang G., Hu X., Liu D. (2011). Duck egg-drop syndrome caused by BYD virus, a new Tembusu-related flavivirus. PLoS One.

[2] Yan P., Zhao Y., Zhang X., Xu D., Dai X., Teng Q., Yan L., Zhou J., Ji X., Zhang S. (2011). An infectious disease of ducks caused by a newly emerged Tembusu virus strain in mainland China. Virology.

[3] Yu K., Sheng Z.Z., Huang B., Ma X., Li Y., Yuan X., Qin Z., Wang D., Chakravarty S., Li F. (2013). Structural, antigenic, and evolutionary characterizations of the envelope protein of newly emerging Duck Tembusu Virus. PLoS One.

[4] Li Y., Huang B., Ma X., Wu J., Li F., Ai W., Song M., Yang H. (2009). Molecular characterization of the genome of duck enteritis virus. Virology.

[5] Liu J., Chen P., Jiang Y., Wu L., Zeng X., Tian G., Ge J., Kawaoka Y., Bu Z, Chen H. (2011). A duck enteritis virus-vectored bivalent live vaccine provides fast and complete protection against H5N1 avian influenza virus infection in ducks. J. Virol..

[6] Liu J., Chen P., Jiang Y., Deng G., Shi J., Wu L., Lin Y., Bu Z., Chen H. (2013). Recombinant duck enteritis virus works as a single-dose vaccine in broilers providing rapid protection against H5N1 influenza infection. Antivir. Res..

[7] Li M.Z., Elledge S.J. (2005). MAGIC, an *in vivo* genetic method for the rapid construction of recombinant DNA molecules. Nat. Genet..

[8] Tan R., Li C., Jiang S., Ma L. (2006). A novel and simple method for construction of recombinant adenoviruses. Nucl. Acids Res..

[9] Wang Z.W., Sarmento L., Wang Y., Li X.Q., Dhingra V., Tseggai T., Jiang B., Fu Z.F. (2005). Attenuated rabies virus activates, while pathogenic rabies virus evades, the host innate immune responses in the central nervous system. J. Virol..

[10] Wang J., Osterrieder N. (2011). Generation of an infectious clone of duck enteritis virus (DEV) and of a vectored DEV expressing hemagglutinin of H5N1 avian influenza virus. Virus Res..

[11] Metcalf W.W., Jiang W., Wanner B.L. (1994). Use of the rep technique for allele replacement to construct new Escherichia coli hosts for maintenance of R6K gamma origin plasmids at different copy numbers. Gene.

[12] Hirt B. (1967). Selective extraction of polyoma DNA from infected mouse cell cultures. J. Mol. Biol..

[13] Li X., Li G., Teng Q., Yu L., Wu X., Li Z. (2013). Development of a blocking ELISA for detection of serum neutralizing antibodies against newly emerged duck Tembusu virus. PLoS One.

[14] Hu Y., Liu X., Zou Z., Jin M. (2013). Glycoprotein C plays a role in the adsorption of duck enteritis virus to chicken embryo fibroblasts cells and in infectivity. Virus Res..

[15] Adler H., Messerle M., Wagner M., Koszinowski U.H. (2000). Cloning and mutagenesis of the murine gammaherpesvirus 68 genome as an infectious bacterial artificial chromosome. J. Virol..

[16] Li S., Zhang L., Wang Y., Wang S., Sun H., Su W., He W., Han B., Su J. (2013). An infectious full-length cDNA clone of duck Tembusu virus, a newly emerging flavivirus causing duck egg drop syndrome in China. Virus Res..

[17] Tang Y., Gao X., Diao Y., Feng Q., Chen H., Liu X., Ge P., Yu C. (2013). Tembusu virus in human, China. Transbound. Emerg. Dis..

[18] Li G., Gao X., Xiao Y., Liu S., Peng S., Li X., Shi Y., Zhang Y., Yu L., Wu X. (2014). Development of a live attenuated vaccine candidate against duck Tembusu viral disease. Virology.

[19] Zelnik V., Tyers P., Smith G.D., Liang C., Ross N.L. (1995). Structure and properties of a herpesvirus of turkeys recombinant in which US1, US10 and SORF3 genes have been replaced by a lacZ expression cassette. J. Gen. Virol..

[20] Chen L., Yu B., Hua J., Ye W., Ni Z., Yun T., Deng X., Zhang C. (2013). Construction of a full-length infectious bacterial artificial chromosome clone of duck enteritis virus vaccine strain. Virol. J..

[21] Li Y., Reddy K., Reid S.M., Cox W.J., Brown I.H., Britton P., Nair V., Iqbal M. (2011). Recombinant herpesvirus of turkeys as a vector-based vaccine against highly pathogenic H7N1 avian influenza and Marek’s disease. Vaccine.

[22] Sonoda K., Sakaguchi M., Okamura H., Yokogawa K., Tokunaga E., Tokiyoshi S., Kawaguchi Y., Hirai K. (2000). Development of an effective polyvalent vaccine against both Marek’s and Newcastle diseases based on recombinant Marek’s disease virus type 1 in commercial chickens with maternal antibodies. J. Virol..

