# Supplementary material for: Efficient Strategy to Generate a Vectored Duck Enteritis Virus Delivering Envelope of Duck Tembusu Virus
Source: Viruses. 2014 Jun 20;6(6):2428–43. doi: 10.3390/v6062428 (PMC4074935; doi:10.3390/v6062428)
Supplement: Supplementary File 1 — Supplementary Materials (PDF, 295 KB) [file viruses-06-02428-s001.pdf]

Supplementary Materials

Efficient Strategy to Generate a Vectored Duck Enteritis Virus Delivering Envelope of Duck Tembusu Virus

Zhong Zou, Zhigang Liu and Meilin Jin

Figure S1. Investigation of the genetic stability of vBAC-C-KCE. The numbers represent the passages of the vBAC-C-KCE (400×). Red fluorescent protein as a selection marker was observed under florescence microscopy.

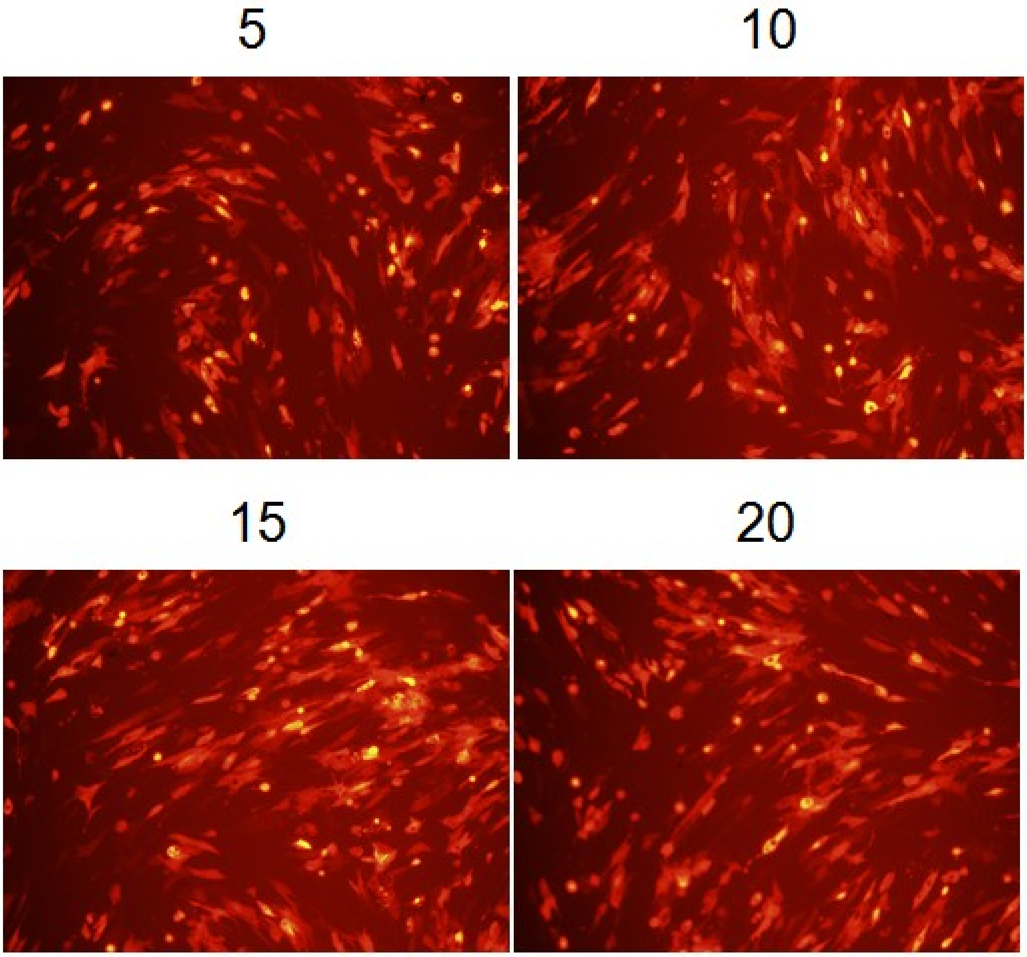

Figure S2. Comparison of the growth between C-KCE, vBAC-C-CKE and C-KCE-E. (**A**) Multi-step growth kinetics of C-KCE-E, vBAC-C-CKE and C-KCE-E in CEFs. Data were shown for the different time points after infection with a multiplicity of infection of 0.01. Shown are the means of virus titers as determined in triplicate; standard deviations are shown with the error bars. (**B**) Plaque size measurement of C-KCE, vBAC-C-CKE and C-KCE-E in CEFs. The means and standard deviations of the area of 100 plaques of both viruses were measured with Image J software. The mean of the plaque size of C-KCE was set at 100%. Standard deviations are shown with the error bars. The multi-step growth curves and plaque size were determined by three independent experiments.

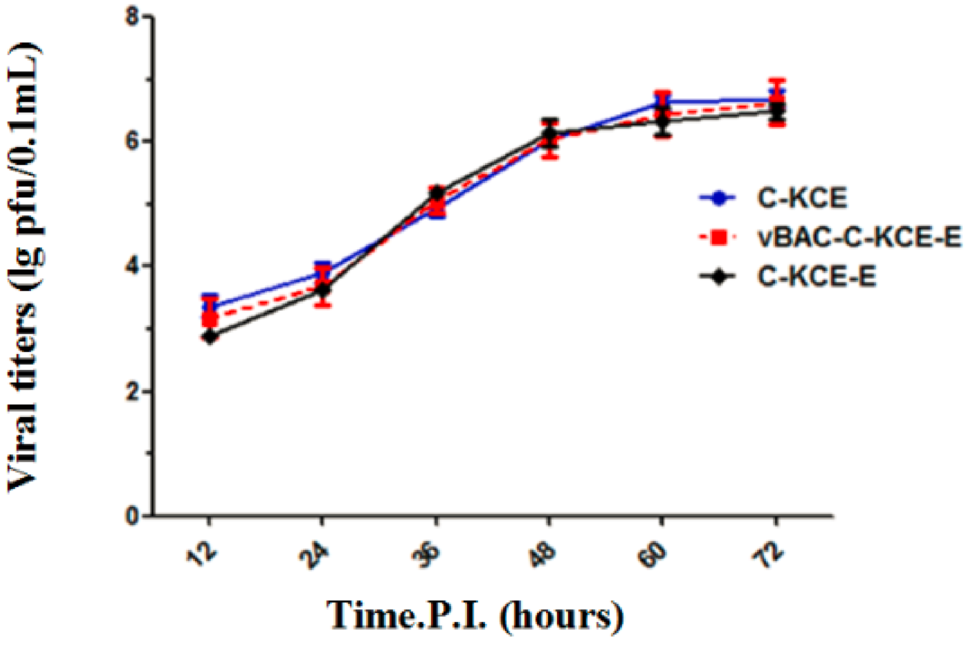

(**A**)

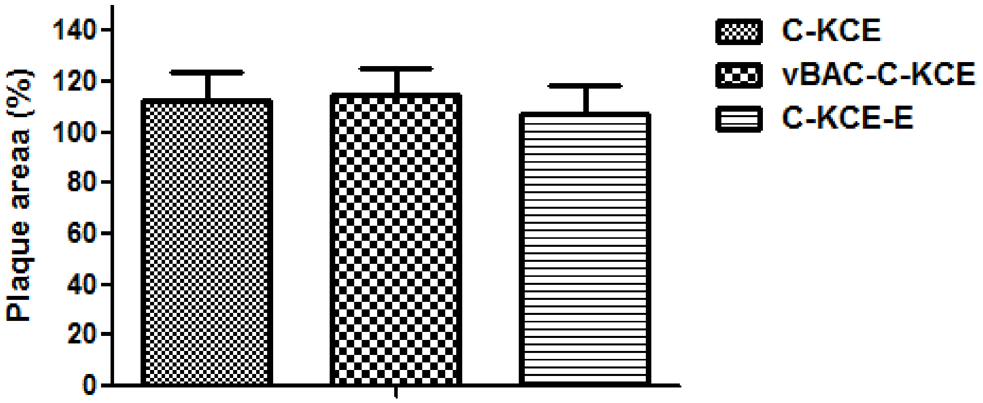

(**B**)
